# Characterization of Ethiopian Wheat Germplasm for Resistance to Four Puccinia *graminis* f. sp. *gtritici* Races Facilitated by Single-Race Nurseries

**DOI:** 10.1094/PDIS-07-18-1243-RE

**Published:** 2019-07-29

**Authors:** Bekele Hundie, Bedada Girma, Zerihun Tadesse, Erena Edae, Pablo Olivera, Endale Hailu Abera, Worku Denbel Bulbula, Bekele Abeyo, Ayele Badebo, Gordon Cisar, Gina Brown-Guedira, Sam Gale, Yue Jin, Matthew N. Rouse

**Affiliations:** 1 Kulumsa Agricultural Research Center, Ethiopian Institute of Agricultural Research, Kulumsa, Ethiopia; 2 Department of Plant Pathology, University of Minnesota, St. Paul, MN 55108, U.S.A; 3 Ambo Plant Protection Research Center, Ethiopian Institute of Agricultural Research, Ambo, Ethiopia; 4 Debre Zeit Agricultural Research Center, Ethiopian Institute of Agricultural Research, Debre Zeit, Ethiopia; 5 International Maize and Wheat Improvement Center, Addis Ababa, Ethiopia; 6 International Programs of the College of Agriculture and Life Sciences, Cornell University, Ithaca, NY 14853, U.S.A; 7 Plant Science Research Unit, U.S. Department of Agriculture–Agricultural Research Service, Raleigh, NC 27695, U.S.A; 8 Cereal Disease Laboratory, U.S. Department of Agriculture–Agricultural Research Service, St. Paul, MN 55108, U.S.A

**Keywords:** cereals and grains, disease management, field crops, fungi

## Abstract

In Ethiopia, breeding rust resistant wheat cultivars is a priority for wheat production. A stem rust epidemic during 2013 to 2014 on previously resistant cultivar Digalu highlighted the need to determine the effectiveness of wheat lines to multiple races of Puccinia graminis f. sp. tritici in Ethiopia. During 2014 and 2015, we evaluated a total of 97 bread wheat and 14 durum wheat genotypes against four P. graminis f. sp. tritici races at the seedling stage and in single-race field nurseries. Resistance genes were postulated using molecular marker assays. Bread wheat lines were resistant to race JRCQC, the race most virulent to durum wheat. Lines with stem rust resistance gene *Sr24* possessed the most effective resistance to the four races. Only three lines with adult plant resistance possessed resistance effective to the four races comparable with cultivars with Sr24. Although responses of the wheat lines across races were positively correlated, wheat lines were identified that possessed adult plant resistance to race TTKSK but were relatively susceptible to race TKTTF. This study demonstrated the importance of testing wheat lines for response to multiple races of the stem rust pathogen to determine if lines possessed non-race-specific resistance.

Worldwide, wheat (Triticum aestivum L.) is fundamental to food security, providing >21% of the calories and 20% of the protein to >4.5 billion people (Braun et al. 2010). The demand for wheat in developing countries has risen and is expected to increase 60% by 2050 (Lucas 2012). Ethiopia is the most important wheat-growing country in sub-Saharan Africa, with 1.6 million hectares and annual grain production of 4.5 million tons at 2.67 t grain yield per hectare (Central Statistical Agency 2017). However, wheat production is constrained by several factors, including fungal diseases. Rust disease pathogens of wheat, Puccinia striiformis Westend f. sp. tritici Erikss, Puccinia graminis Pers:Pers f. sp. tritici Erikss & E Henn, and Puccinia triticina Erikss, cause some of the most damaging diseases of wheat worldwide (Roelfs et al. 1992). The devastating effects of stem rust epidemics were observed in East Africa (in Kenya in the early 1990s and Ethiopia in 1993) because of a breakdown of the resistance gene Sr36 in cultivar Enkoy (Kebede et al. 1995). P. graminis f. sp. tritici race TTKSK (Ug99) (Pretorius et al. 2000) and race TKTTF (Olivera et al. 2015) have been detected in East Africa. Race TTKSK is virulent to most wheat stem rust resistance genes used in wheat cultivars (Singh et al. 2015). More specifically, race TTKSK possesses unique virulence to Sr31 and Sr38, which are resistance genes widely used in wheat cultivars worldwide (Pretorius et al. 2000). Of the wheat cultivars grown globally, 80 to 90% were reported to be susceptible to TTKSK (Singh et al. 2006). Thirteen races in the TTKSK race group have been reported in Africa, Yemen, and Iran (Newcomb et al. 2016; Singh et al. 2015). Variability within P. graminis f. sp. tritici populations is high in Ethiopia (Admassu and Fekadu 2005; Admassu et al. 2009). Race TTKSK was the most dominant and virulent race in Ethiopia up to 2014 (Hailu and Woldeab 2015). However, other P. graminis f. sp. tritici races, including TKTTF, TRTTF, and JRCQC, have been detected in Ethiopia (Hailu and Woldeab 2015; Olivera et al. 2012, 2015). Race TKTTF, also known as the “Digalu race,” infected the popular bread wheat cultivar Digalu in Ethiopia, causing up to 100% yield losses between 2013 and 2015 (Olivera et al. 2015). Digalu was released in 2005 by Kulumsa Agricultural Research Center (ARC) (Ministry of Agriculture and Natural Resources 2016) and became the most widely cultivated wheat cultivar in Ethiopia starting in 2011 after a 2010 stripe rust epidemic in Ethiopia (Worku 2014). Digalu possessed resistance to race TTKSK conferred by SrTmp but was susceptible to race TKTTF (Abebe et al. 2012; Olivera et al. 2015; Worku et al. 2013). After the severe stem rust outbreak in 2013, race TKTTF became the predominant race in Ethiopia (Olivera et al. 2017). Because Digalu is resistant to race TTKSK, this epidemic highlighted the need to select wheat cultivars with resistance to both race TTKSK and other P. graminis f. sp. triciti races, including TKTTF.

Stem rust has long been managed with resistant cultivars and chemical control in Ethiopia (Badebo et al. 2008; Tesemma and Mohammed 1982) as well as worldwide (McIntosh et al. 1995). However, chemical options to control stem rust are sometimes not accessible because of high cost and lack of supply (Beard et al. 2006). Genetic resistance is the most economically viable method of controlling stem rust. The resistance effect conferred by all-stage resistance genes is usually greater than the resistance effect of adult plant resistance genes. However, all-stage resistance is often not durable, because virulent races of *P*. *graminis* f. sp. *tritici* often evolve to overcome this type of resistance (Jin et al. 2008; Olivera et al. 2015; Pretorius et al. 2000). Abundant wheat germplasm from introductions and local crosses has been screened for reaction to stem rust in Ethiopia, and cultivars with all-stage resistance genes have been released and placed into cultivation to sustain wheat production. Unfortunately, these resistant cultivars were soon overcome by virulent races (Badebo et al. 2008; Huluka et al. 1991; Olivera et al. 2015). The failure of all-stage resistance genes because of virulence diversity in the P. graminis f. sp. tritici population threatens wheat production worldwide and has led researchers to select for adult plant resistance (Singh et al. 2011). The idea behind this strategy is that adult plant resistance would prove more durable. Information on both all-stage and adult plant resistance genes in wheat lines is desirable for informing cultivar deployment and breeding line advancement. However, data on Sr genes in Ethiopian cultivars and advanced lines are scarce, and the field stem rust response of Ethiopian lines is largely unknown.

Therefore, the objectives of this research were to (1) characterize Ethiopian wheat cultivars and advanced germplasm for their response to four P. graminis f. sp. tritici races present in Ethiopia (TKTTF, TTKSK, TRTTF, and JRCQC) and (2) postulate the presence of various stem rust resistance genes in Ethiopian bread wheat cultivars and advanced lines.

## Materials and Methods

P. graminis f. sp. tritici isolates. During 2014, two collections of P. graminis f. sp. tritici were obtained from the Debre Zeit ARC in Ethiopia. In addition, two stem rust collections were made from wheat fields outside Ambo and in the Assasa plain in Ethiopia. We derived one isolate from each collection from single pustules of inoculated plants at the Ambo Plant Protection Research Center, Ethiopia. The four isolates were typed as races TTKSK (isolate P14ETH02-1), TRTTF (33 Wonchi-1), TKTTF (Digalu 1/1 Assasa), and JRCQC (PETH01DZ-2) after testing against the North American stem rust differential set (Jin et al. 2008; Roelfs and Martens 1988). These isolates were used to inoculate the field nurseries. Isolates 04KEN156/04 (TTKSK), 06YEM34-1 (TRTTF), 13ETH18-1 (TKTTF), and 09ETH08-3 (JRCQC) available from previous studies at the USDA-Agricultural Research Service (ARS) Cereal Disease Laboratory (Jin and Singh 2006; Olivera et al. 2012, 2015) were used in seedling assays completed at the Cereal Disease Laboratory.

Plant materials. The stem rust susceptible spring wheat line LMPG-6 and eight near-isogenic lines (NILs) with different stem rust resistance (Sr) genes in the LMPG genetic background were obtained from the USDA-ARS Cereal Disease Laboratory (Knott 1990). In addition, three two-gene combination lines in the LMPG genetic background were obtained from the USDA-ARS Cereal Disease Laboratory (Newcomb et al. 2016). These NILs were used to monitor the P. graminis f. sp. tritici races present in each of the field nurseries and are described in Supplementary Table S1.

A total of 111 genotypes were tested: 24 Ethiopian bread wheat cultivars, 23 advanced bread wheat breeding lines from the Ethiopian Institute of Agricultural Research (EIAR), 50 advanced bread wheat breeding lines from the International Maize and Wheat Improvement Center (CIMMYT), nine Ethiopian durum wheat (Triticum turgidum ssp. durum [Desf] Husnot) cultivars, and five advanced durum wheat breeding lines from either CIMMYT or the International Center for Agricultural Research in Dry Areas. Seeds of the bread wheat cultivars and breeding lines were obtained from the Kulumsa ARC, the Sinana ARC, the Adet ARC, and the Debre Birhan ARC. The durum wheat cultivars and breeding lines were obtained from the Debre Zeit and Sinana ARCs.

Five Ethiopian wheat cultivars were used as repeated checks in the field nurseries. These check lines included four bread wheat cultivars: cultivars Kubsa, Digalu, Danda’a, and Kingbird (Ministry of Agriculture and Natural Resources 2016). The durum wheat cultivar Arendeto (DZ04-118) was also included as a check.

A total of five wheat cultivars were used as spreader rows in the field nurseries. Cultivar PBW343 possesses Sr31, and it is susceptible to race TTKSK but not to races TRTTF, TKTTF, or JRCQC. PBW343 was obtained from the Kulumsa ARC and used as a spreader in the TTKSK field nursery. Digalu is susceptible to race TKTTF but not to TTKSK, TRTTF, or JRCQC. Digalu was used as a spreader in the TKTTF field nursery. Cultivar Laketch is susceptible to race TRTTF but not to the other races, and it was used as a spreader in the TRTTF field nursery. Cultivar ST464 possesses Sr13 (Zhang et al. 2017) and is relatively susceptible to race JRCQC (Olivera et al. 2012) but not to the other races; it was used as a spreader in the JRCQC field nursery. Seeds of PBW343 and Laketch were obtained from the Kulumsa ARC. ST464 was obtained from the USDA-ARS Cereal Disease Laboratory. During the 2014 field season, limited seed of Laketch was available, and therefore, a mixture of Laketch and Kubsa was used as a spreader in the TRTTF nursery.

Seedling infection-type assays. Seed of the cultivars, breeding lines, NILs, and check lines were assessed for seedling infection types (ITs) at the USDA-ARS Cereal Disease Laboratory except for the durum lines, which were not permitted to be exported from Ethiopia. The lines were assessed in two replications each for reactions to races TTKSK, TRTTF, TKTTF, and JRCQC in addition to three other races within the TTKSK race group (TTKST, isolate 06KEN19v3; TTKTT, 14KEN58-1; and TTTSK, 07KEN24-4). Lines were planted in plastic pots (6.7-cm width × 6.7-cm length × 5.7-cm height) filled with vermiculite (Sun Gro Horticulture). In each pot, four lines were planted with five seeds per line per replication. After planting, trays were incubated in a greenhouse maintained at 19 to 22 °C with a photoperiod of 16 h manipulated by supplemental lighting. The seedling plants were inoculated when the primary leaf had fully emerged and the second leaf was beginning to grow, which was ˜10 days after planting. Isolates were derived from single pustules and retrieved from storage at –80°C (Rouse et al. 2011). Urediniospores were heat shocked by submergence of plastic bags containing the isolates within a water bath set at 45°C for 15 min and suspended in lightweight mineral oil (Soltrol 70; ConocoPhillips Inc.) in preparation for inoculation. A total of 0.75 ml of oil was added to individual gelatin capsules each including 14 mg of urediniospores. One gelatin capsule was used to inoculate 48 wheat lines (240 seedling plants total per capsule) using a custom rust inoculator (St. Paul Machine Shop, University of Minnesota) pressurized by an air pump (30 kPa). Immediately after inoculation, the oil was allowed to evaporate for 15 min, and inoculated plants were subsequently placed into dew chambers maintained at 22°C, where ultrasonic humidifiers (V5100NS; Vicks) supplied mist to the chamber for 2 min every 15 min for ˜16 h without light. Next, 400-W high-pressure sodium vapor lamps (LR217718; Kavita Canada Inc.) were turned on above the dew chambers that possessed a transparent plastic roof, allowing light penetration to the plants. After 2 h, the doors of the dew chambers were opened, and the plants were moved to a greenhouse maintained at 19 to 22 °C with a photoperiod of 16 h manipulated by supplemental lighting. Fourteen days after inoculation, ITs were recorded according to the 0 to 4 scale developed by Stakman et al. (1962), where 0, ;, 1, and 2 indicate resistance and 3 and 4 indicate susceptibility. Relatively smaller and larger pustule sizes for each IT class were noted with - and + symbols, respectively. When two or more ITs were present on the same leaf, all ITs were recorded with the most frequent IT listed first. When a line was heterogeneous for IT, a / symbol was used to separate ITs recorded for different plants.

Single-race field nurseries. At the Kulumsa ARC, four singlerace nurseries were maintained in June through October of 2014 and 2015. Each nursery consisted of a different field separated by at least 200 m from all other stem rust nurseries and consisted of plots, spreader rows, and borders. Plots were composed of two 0.5-m rows spaced 20 cm apart and planted with 1.5 g of seed. The distance between plots was 0.4 m. Spreader rows were sown perpendicular to the plots in the middle of 1-m alleys between adjacent rows of plots. Surrounding each nursery, 2 m of border was planted with three rows of oats (1 m; inside) and three rows of rapeseed (1m; outside). The borders and spreaders were planted 1 to 2 weeks before the entries were planted. The test entries were planted in each nursery in an augmented design, with six replications of the five check lines. In addition, two replications of the NILs were planted in each nursery.

Urediniospores of pure races TTKSK, TRTTF, TKTTF, and JRCQC were increased in isolation inside plastic chambers in a greenhouse on seedlings of susceptible wheat line McNair 701 (CItr 15288) grown in plastic pots at a rate of 20 plants per pot. At seedling emergence (4 days after planting), the McNair 701 plants were treated with a maleic hydrazide solution (25 ml of a 0.33 g maleic hydrazide per 1 liter water mixture were supplied to each pot) to limit plant growth to one or two leaves and increase pathogen fitness. Urediniospores were collected from the sporulating pustules of inoculated plants starting 14 days after inoculation using a custom rust collector (St. Paul Machine Shop, University of Minnesota) attached to an air pump (separate air pumps were used for urediniospore inoculations and collections in both the United States and Ethiopia). Urediniospores were delivered in 25-ml glass tubes from the Ambo Plant Protection Research Center to the Kulumsa ARC for inoculation of the nurseries. A total of 1 g of urediniospores of each isolate were suspended in 1 liter of a lightweight mineral oil (Soltrol 70; ConocoPhillips Inc.) and sprayed onto the spreader rows of each nursery using an ultralow-volume sprayer with a single nozzle (ULVA+; Micron Sprayers). A different sprayer was used for each nursery, and spreaders were inoculated two to three times each season in between the stem elongation and flowering growth stages. Also, spreader rows were inoculated with a suspension of 0.5 g urediniospores, 500 ml water, and 1 drop Tween 20 surfactant. Disposable syringes (10 ml) were used to inject 1 ml of the spore suspension in ˜500 stems of the wheat line planted as a spreader spaced out at ˜1 stem per meter at the stem elongation stage. Personnel entering the nurseries wore disposable Tyvek suits, goggles, and gloves.

During each screening season, disease severity (DS) and infection response (IR) were visually assessed four times starting at the onset of disease. Final disease ratings were made at the hard dough growth stage. Disease severity was assessed on the 0 to 100% modified Cobb scale (Peterson et al. 1948), and IR was classified into categories of resistant, moderately resistant, moderately susceptible, and susceptible based on pustule size and amount of chlorosis (Roelfs et al. 1992). When more than one IR was observed in the same plot, all IRs were recorded with the most frequent IR listed first. The coefficient of infection (COI) was calculated as the product of the disease severity value and a linearized IR score according to Gao et al. (2016). Area under the disease progress curve (AUDPC) was calculated according to Wilcoxson et al. (1975) for each wheat genotype.

Molecular marker assays. Tissue was harvested from the second leaf of seedlings of the Ethiopian wheat germplasm, checks, and NILs and delivered to the USDA-ARS Plant Science Research Unit in Raleigh, North Carolina for DNA extraction and molecular marker assessment. Molecular markers linked to Sr2, Sr22, Sr24, Sr25, Sr26, Sr31, Sr35, Sr36, Sr38/Lr37/Yr17, Sr57/Lr34/Yr18, and Sr1RS^amigo^ were assessed on the wheat lines. Marker wMAS000005 was assayed to predict the presence or absence of Sr2. wMAS000005 is a Kompetitive Allele-Specific PCR (KASP) assay marker developed from information in Mago et al. (2011) and described on the MASwheat website (https://maswheat.ucdavis.edu). KASP markers indicative of introgression segments possessing Sr38, Sr22, and Sr26 were developed based on sequence of markers reported in Helguera et al. (2003), Mago et al. (2005), and Periyannan et al. (2011). SSR marker Xbarc71 was assayed to predict *Sr24* (Mago et al. 2005; Somers et al. 2004). Marker Gb was assayed to predict Sr25 (Yu et al. 2010). Marker scm9 (Kofler et al. 2008) in combination with KASP assays developed from source sequences of Illumina iSelect SNPs *wsnp*_ Ra_c6474_11298858 and *wsnp*_JD_c5757_6915127 were assayed to predict Sr1RS^Amigo^. Marker scm9 (Kofler et al. 2008) in combination with a KASP assay developed from *wsnp*_Ex_rep_c66282_ 64438053 was assayed to predict Sr31. Gene Sr35 was predicted using a dominant KASP assay designed from the sequence of the cloned gene (Saintenac et al. 2013). Markers Xwmc477 (Tsilo et al. 2008) and wMAS000015 were used to assay for Sr36. wMAS000015 is described on the MASwheat website. Markers wMAS000003 and Lr34-Jagger were assayed for Lr34/Sr57. Both KASP assay markers are described on the MASwheat website and derived from information published in Lagudah et al. (2009).

Data analyses. Analysis of variance was conducted on AUDPC, COI, and severity (disease parameters) to determine resistance differences among the wheat genotypes. The analysis was conducted using PROC MIXED in SAS (Version 9.3; SAS Institute, Inc.) following mixed model procedures for an augmented design, where blocks and check cultivars were considered as fixed effects and the effect of tested lines was considered as random. A correlation coefficient was used to estimate the relationship between the different disease parameters: AUDPC, COI, and disease severity.

## Results

Plant materials. The seedling infection-type assays and molecular marker data confirmed the expected genetic compositions of the NIL lines (Table 1). The marker and seedling data validated the expected stem rust resistance gene content in the NILs. The seed stocks for the Sr36 checks were heterogeneous as revealed by both seedling infection types and marker assays. AUDPC values indicated that disease was most severe in the TTKSK and TKTTF treatments. Comparing AUDPC values among the NILs revealed that the nurseries were primarily composed of the P. *graminis* f. sp. *tritici* races inoculated at each nursery. NIL DK42 with Sr31 showed susceptibility only in the race TTKSK nursery. DK37 with Sr11 showed resistance only in the race TKTTF nursery. DK4 with Sr8a showed resistance only in the TRTTF nursery. DK10 with Sr13 displayed substantially lower responses compared with susceptible LMPG-6 in each nursery, except the JRCQC nursery. DK15, CDLSr24Sr31, and CDLSr24Sr36, each with Sr24, were resistant in all four nurseries. In summary, AUDPC values among the differential wheat lines demonstrated that wheat lines in each single-race field nursery were exposed to a single P. *graminis* f. sp. *tritici* race.

**Table 1:** Average area under the disease progress curve (AUDPC) values across four single-race nurseries, seedling infection types in response to inoculation using four races of *Puccinia graminis* f. sp. *tritici*, and molecular marker genotypes of near-isogenic lines with stem rust resistance genes in susceptible background line LMPG-6

Line	2014–2015 Average AUDPC				Seedling infection type				Molecular marker		
	TTKSK	TKTTF	TRTTF	JRCQC	TTKSK	TKTTF	TRTTF	JRCQC	*Sr31*	*Sr24*	*Sr36*
LMPG-6	768.4	749.9	478.7	608.0	3+	3+	3+	3+	–	–	–
DK1-Sr5	640.3	580.4	404.2	242.1	3+	3+	3+	0;/0;3/33+	–	–	–
DK4-Sr8a	660.8	456.5	256.0	425.8	3+	33+/3+	2–/2	2–/22–	–	–	–
DK8-Sr9e	549.6	649.7	301.0	617.4	3+	3+	32+/33+	3+	–	–	–
DK37-Sr11	752.3	347.5	375.1	544.5	3+	12–/2–/3+	3+	3+/33+	–	–	–
DK10-Sr13	378.9	525.4	334.8	572.5	2/22–	2+3/32+/33+	33+	33+/3/2+3	–	–	–
DK15-Sr24	223.6	198.7	160.9	149.6	2–/;2–	2–/;2–	2–	2–	–	+	–
DK42-Sr31	717.2	330.4	248.8	354.7	3+	2–/3+	2–	2–	+	–	–
DK22-Sr36	87.0	360.8	167.8	58.5	0;/3	3+	3+	0;/0;3/3+	–	–	+
CDLSr24Sr31	240.3	188.1	127.7	160.8	2–/;2–	2–/;2–	2–	2–/;2–	+	+	–
CDLSr24Sr36	73.7	152.0	101.8	32.0	0;	2–/;2–	2–	0;	–	+	+ het
CDLSr31Sr36	173.6	165.4	125.3	62.9	0;/0;1/3/33+	2–/3+	2–/3+/0;/2;	0;/2–/0;2–/3+	+	–	+ het

Seedling infection-type assays. The check cultivars had variable reactions to the four races at the seedling plant stage (Table 2). Kingbird was consistently resistant in each of the single-race nurseries relative to the other checks, and Kubsa was susceptible except to race JRCQC (Table 2). Of all 97 bread wheat lines, including the check cultivars, 35, 67, 45, and 98% were resistant to races TTKSK, TRTTF, TKTTF, and JRCQC, respectively (Supplementary Table S2). None of the check cultivars were either susceptible or resistant to all four P. graminis f. sp. tritici races. Generally, the checks were susceptible to races TTKSK, TRTTF, and TKTTF but resistant to race JRCQC. Exceptions included Arendeto being resistant to race TRTTF but susceptible to race JRCQC and Digalu being resistant to all races except TKTTF. Danda’a and Kubsa displayed intermediate responses to race TKTTF, whereas Kingbird displayed an intermediate response to race TRTTF. A total of 24% of the cultivars and lines from CIMMYT and EIAR were resistant to all four races at the seedling stage. None of the lines were susceptible to all four races as evaluated in the seedling stage. However, 6.5% of the lines were seedling susceptible to the three races TTKSK, TRTTF, and TKTTF. These lines included five from CIMMYT and cultivar Gassay. A total of 91% of the lines with seedling resistance to race TTKSK were susceptible or less resistant to race TTKSK variants with *Sr24* and/or SrTmp virulence (Supplementary Table S3). This indicates that most lines with seedling resistance to race TTKSK possessed *Sr24* or SrTmp.

**Table 2:** Average area under the disease progress curve (AUDPC) values across four single-race nurseries, seedling infection types in response to four races of *Puccinia graminis* f. sp. *tritici*, and molecular marker genotypes of the augmented check cultivars

Cultivars	2014–2015 Average AUDPC				Seedling infection type				Molecular marker		
	TTKSK	TKTTF	TRTTF	JRCQC	TTKSK	TKTTF	TRTTF	JRCQC	*Sr2*	*Sr57*	*Sr38*
Arendeto	788.7 cd^y^	903.6 b	469.2 b	875.0 b	3+/33+	3+/33+	2 to 22–^z^	33+	–	–	–
Danda’a	635.9 bc	630.7 b	364.0 ab	209.1 a	3+/33+	2+3/32+	3+/33+	0;1 to 2–	–	–	–
Digalu	431.1 ab	808.0 b	409.7 b	356.7 a	2/22–	3+/33+	22+/2/2+3	0; to 2	–	–	+
Kingbird	225.2 a	129.5 a	57.5 a	51.0 a	;3– to 33+	;3 to 3+	;12– to 3	0;1 to 2–	+	+	–
Kubsa	1,003.7 d	812.0 b	625.8 b	301.7 a	3+/33+	2+3 to 3+	3+/33+	;2– to 2–	–	–	–

y Significant differences (*P* < 0.05) among lines within single-race nurseries based on least squares means in analysis of variance are denoted by different letters.

z Because checks were tested for seedling infection type in >12 assays, the range of infection types observed is sometimes given.

**Fig. 1:**
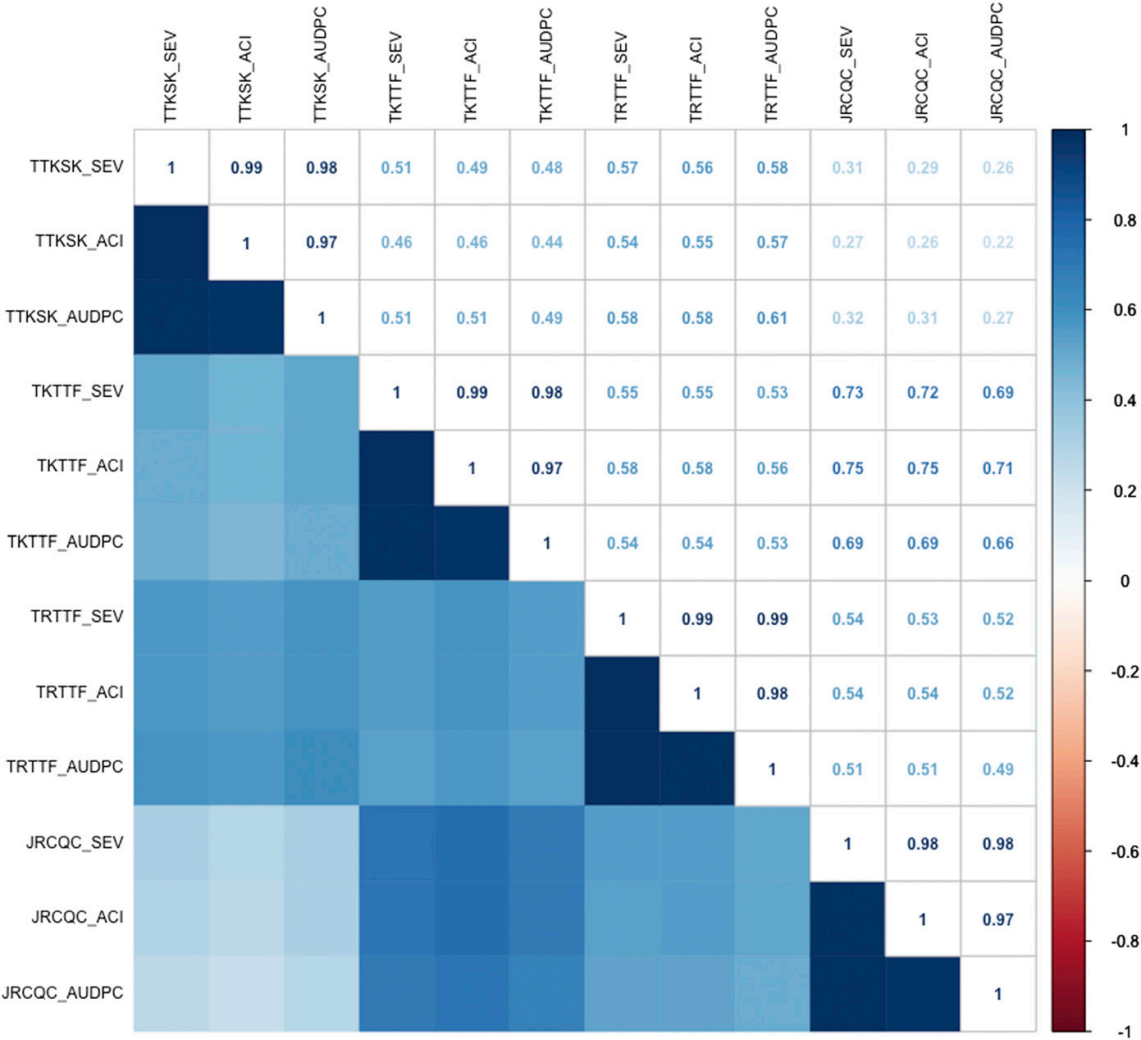
Correlation coefficients among disease severity (SEV), average coefficient of infection (ACI), and area under the disease progress curve (AUDPC) for the average values for each of the four single-race nurseries during 2014 and 2015.

Single-race field nurseries. As expected, disease severity, COI, and AUDPC were highly correlated within each single-race nursery (coefficients ranged from 0.97 to 0.99) (Fig. 1). Among the various nurseries, correlations were positive and ranged from 0.22 (low) to 0.75 (high) (Fig. 1). The lowest correlations of disease indices were between races TTKSK and JRCQC. This result demonstrates that response of wheat lines to relatively avirulent JRCQC was least comparable with virulent race TTKSK. The highest correlations were between races TKTTF and JRCQC. This result is somewhat unexpected, because TKTTF is virulent compared with JRCQC. Perhaps the two races share a common avirulence to a wheat stem rust resistance gene that may be present in the lines, such as Sr7a. The effects of checks and lines were significant for each single-race nursery for each of the three disease parameters (Tables 3 and 4). The effect of year (2014 versus 2015) was significant for eight of the 12 possible combinations of race and disease parameter. The effect of blocks was not significant (Tables 3 and 4). The absence of significant block effects indicates that disease pressure was even within each nursery, which provides confidence in interpreting data on the germplasm tested.

**Table 3:** Analysis of variance table highlighting levels of significance observed among various parameters in the inoculated TTKSK and TKTTF single-race nurseries during 2014 and 2015

Parameter	Degrees of freedom	TTKSK			TKTTF		
		Severity	COIw	AUDPC	Severity	COI	AUDPC
Year	1	393.5^x^	1,352.4^y^	9,415,668^y^	172.9^z^	63.3^z^	11,104,586^y^
Block	6	73.7^z^	75.8^z^	36,763^z^	18.7^z^	16.7^z^	9,781.8^z^
Checks	4	2,479.3^y^	2,697.4^y^	1,194,052^y^	2,603.7^y^	2,972.9^y^	1,257,771^y^
Lines	165	288.9^y^	311.7^y^	151,660^y^	192.2^y^	202.7^y^	115,602^y^
Residual	190	84.2	82.7	27,581	69.2	66	34,181

w AUDPC, area under the disease progress curve; COI, coefficient of infection.

x Significantly different at 0.05.

y Significantly different at <0.001.

z NS, not significantly different.

**Table 4:** Analysis of variance table highlighting levels of significance observed among various parameters in the inoculated TRTTF and JRCQC single-race nurseries during 2014 and 2015

		TRTTF			JRCQC		
Parameter	Degrees of freedom	Severity	COI^x^	AUDPC	Severity	COI	AUDPC
Year	1	3,114.7y	2,424.6^y^	10,279,914^y^	89.9z	14.3^z^	4,566,954^y^
Block	6	27.7^z^	28.6^z^	19,722^z^	17.8^z^	17.2^z^	18,508^z^
Checks	4	844.3^y^	924.3^y^	563,218^y^	2,374.1^y^	2,614.6^y^	1,257,434^y^
Lines	165	96.2^y^	102.5^y^	73,663^y^	162.8^y^	176.3^y^	102,732^y^
Residual	190	27	27.6	27,664	31.9	31.7	28,415

x AUDPC, area under the disease progress curve; COI, coefficient of infection.

y Significantly different at <0.001.

z NS, not significantly different.

**Fig. 2:**
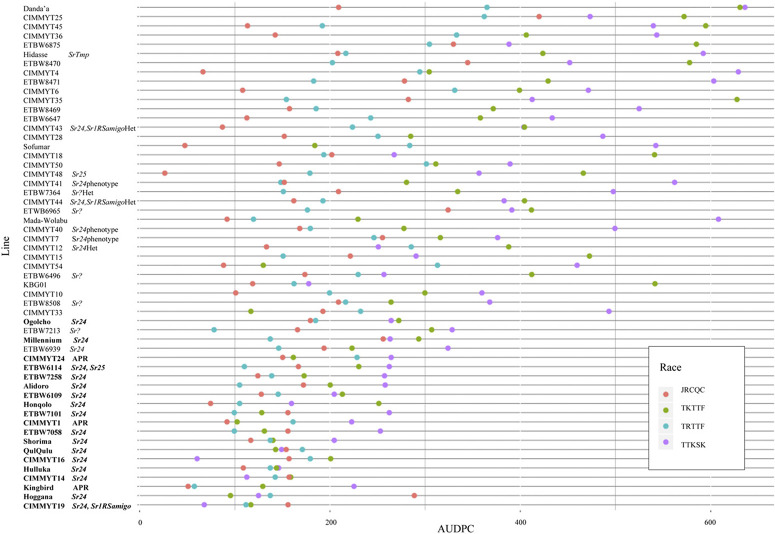
Average area under the disease progress curve (AUDPC) values of wheat cultivar Danda’a and the 54 wheat lines displaying average AUDPC values less than Danda’a in response to *Puccinia graminis* f. sp. *tritici* races TTKSK, TKTTF, TRTTF, and JRCQC. Stem rust resistance genes effective to TTKSK are denoted next to line names. Names in bold font are those lines that showed AUDPC values for TKTTF and TTKSK significantly less than that of Danda’a based on least squares means. *Sr*? indicates an unknown seedling resistance gene effective to race TTKSK.

Overall, disease was greater in the TTKSK and TKTTF nurseries (Supplementary Table S4). Data on the check cultivars were difficult to interpret, because we lacked a true susceptible check cultivar (each check cultivar was seedling resistant to at least one race). Danda’a had consistently intermediate responses in between those of Kingbird and Kubsa for races TTKSK, TKTTF, and TRTTF. We chose to emphasize disease resistance relative to Danda’a, because this line can be considered to possess intermediate resistance, but the resistance level is insufficient. For bread wheat, nearly all lines were resistant to JRCQC in the field. A total of 54 of the 97 bread wheat lines possessed average AUDPC values less than that of Danda’a in response to TTKSK, TKTTF, and TRTTF (Fig. 2). Using least squares means, we identified just 19 lines that possessed significantly lower AUDPC compared with Danda’a in the TTKSK and TKTTF nurseries (in bold font in Fig. 2). A total of 84% of these lines possessed Sr24, and 16% possessed adult plant resistance. The three lines with adult plant resistance were Kingbird, CIMMYT 1, and CIMMYT 24. However, CIMMYT 24 was heterogeneous for seedling resistance to race TTKSK in one replication. Because the response of Danda’a to race TRTTF was quite low, only one breeding line (ETBW7213) was significantly more resistant than Danda’a in response to race TRTTF.

The AUDPC of the 13 durum wheat lines assessed was lower than that of older durum cultivar Arendeto in all four nurseries (Table 5). In general, the durum lines were resistant compared with the bread wheat lines in response to races TTKSK, TKTTF, and TRTTF, but the durum lines were more susceptible relative to bread wheat lines for the Sr13b-virulent race JRCQC. Among the durum lines, cultivars Boohai and Kilinto possessed the greatest resistance to race JRCQC and were intermediate in resistance to the other races as well. Results of assessing the stem rust response of the limited number of durum cultivars and breeding lines tested indicated that race JRCQC is especially virulent to durum wheat compared with the other P. graminis f. sp. tritici races tested. Testing numerous additional durum wheat lines against race JRCQC, especially cultivars and advanced breeding lines, is needed to identify resistant durum lines.

**Table 5:** Average area under the disease progress curve (AUDPC) observed during 2014 and 2015 for the durum wheat lines included in the four single-race nurseries

Line or cultivar		2014–2015 Average AUDPC		
	TTKSK	TKTTF TRTTF		JRCQC
Arendeto	788.7	903.6	469.2	875.0
EIAR 1	247.5^z^	204.3^z^	214.5	384.5^z^
EIAR 2	229.2^z^	201.8^z^	245.7	395.5^z^
EIAR 3	401.8^z^	452.3^z^	193.8	369.5^z^
EIAR 4	167.4^z^	144.8^z^	216.3	306.9^z^
EIAR 5	121.4^z^	178.3^z^	208.0	344.3^z^
Bakalcha	227.1^z^	193.6^z^	206.7	268.6^z^
Boohai	118.3^z^	190.9^z^	223.8	80.5^z^
Cocorit-71	180.9^z^	177.2^z^	99.2^z^	291.0^z^
Ejersa	405.9^z^	381.4^z^	224.5	479.7^z^
Kilinto	337.0^z^	313.2^z^	237.7	100.2^z^
Mangudo	232.7^z^	166.0^z^	210.9	272.8^z^
Toltu	339.6^z^	236.2^z^	180.7^z^	476.8^z^
Yerer	152.4^z^	237.0^z^	170.1^z^	315.3^z^

z Significantly different based on least squares means compared with augmented check line Arendeto at 0.05.

Molecular marker assays. Of the 97 bread wheat lines (including checks but not NILs), 13, 21, 25, 13, 3, 2, and 31% possessed Sr2, Sr57/Lr34, Sr24, Sr31, Sr1RS^amigo^, Sr25, and Sr38, respectively, based on molecular marker genotypes (Supplementary Table S5). Genes Sr22, Sr26, Sr35, Sr36, Sr42, and Sr55/Lr67 were not detected. Lines with *Sr24* were resistant as seedlings to all four races except for CIMMYT 12, CIMMYT 43, and CIMMYT 44, which gave susceptible responses to at least one Sr24-avirulent race in at least one replication, indicating that these lines are likely heterogeneous for Sr24. Lines ETBW8470 and ETBW6875 possessed the resistant haplotype of the *Sr24* marker but were susceptible to race TKTTF and/or TTKSK, indicating that these are likely false positive for Sr24. CIMMYT 40 and CIMMYT 41 displayed an Sr24-characteristic seedling infection-type pattern (resistant to all races except the Sr24-virulent TTKST and TTKTT) but lacked the resistant haplotype of the *Sr24* marker. These two lines were also more susceptible in the field compared with the lines with both the *Sr24* resistant allele and infection-type pattern. Only Kingbird, cultivar Sofumar, and line ETBW7364 possessed both adult plant resistance genes

Sr2 and Sr57/Lr34, with the latter being heterogeneous for Sr57/ a combination of two or more effective seedling resistance genes: Lr34. Although Sr38 was observed at the highest frequency of ETBW6114 (Sr24, Sr25), CIMMYT 19 (Sr1RS^amigo^, Sr24), CIM-any of the genes, Sr38 does not confer resistance to TTKSK, MYT 43 (Sr24, Sr1RS^amigo^, Sr31 heterogeneous), and CIMMYT TKTTF, or TRTTF. Several breeding lines were identified with 44 (Sr1RS^amigo^, *Sr24* heterogeneous). Additional tests are needed to validate the genetic constitution of selections of CIMMYT 43 and CIMMYT 44.

## Discussion

The most immediate result of our study is the identification of wheat germplasm with resistance to multiple races of P. graminis f. sp. tritici in Ethiopia. We identified 19 bread wheat lines that possessed significantly greater resistance than Danda’a, the cultivar previously reported to contain a level of resistance to the local stem rust races. Unfortunately, the resistance in 16 of these lines is conferred by Sr24. Virulence to *Sr24* is common in the P. graminis f. sp. tritici population in Kenya (Newcomb et al. 2016). If *Sr24* virulence was to predominate in Ethiopia, many, if not all, of these 16 lines could become susceptible over time. In addition, we identified only three lines with adult plant resistance that are significantly more resistant than Danda’a as measured by AUDPC.

Our marker and seedling data suggest a low frequency of effective stem rust resistance genes in Ethiopian germplasm. Genes, such as Sr22, Sr25, and Sr26, are effective to the races considered in this study and could be increased in frequency in Ethiopian germplasm to improve resistance. For adult plant resistance genes, increasing the frequency of lines with combinations of genes Sr2 and Sr57/Lr34 may ultimately improve resistance. However, the Sr2 and Sr57/Lr34 combination alone may not result in adequate resistance, because Sofumar with this combination did not display significantly lower AUDPC compared with Danda’a in response to race TTKSK. Our data revealed several examples of lines carrying Sr2 but with a greater AUDPC to TTKSK than Danda’a (ETBW6696, Sofumar, and Tay).

Wheat lines, including ETBW8508, and ETBW7213, possessed seedling resistance to race TTKSK that did not correspond to resistance genes postulated by the molecular markers. These lines may possess TTKSK-effective resistance gene Sr9h, Sr13, or SrND643 (Basnet et al. 2015; Rouse et al. 2014a). The resistant seedling reactions of the wheat lines in this study to TKTTF, TRTTF, and JRCQC warrant additional screening. Previously, Sr11 was reported to be effective to race TKTTF and present in Ethiopian wheat germplasm (Nirmala et al. 2016). Association mapping studies have confirmed the presence of Sr7a, which confers resistance to race TKTTF, and Sr8a, which confers resistance to TRTTF, in conventional germ-plasm (Bajgain et al. 2015; Gao et al. 2017; Mihalyov et al. 2017). Although nearly all of the bread wheat lines were resistant to race JRCQC, the genes specifically responsible for conferring this resistance are unknown.

Two lines produced susceptible seedling reactions to race TTKSK and resistant field responses to race TTKSK (indicating adult plant resistance) but susceptible field responses to race TKTTF (KBG-01 and CIMMYT 18). This finding highlights the value of testing for field response to multiple stem rust pathogen races in separate single-race nurseries. For example, if data from only a TTKSK race group field experiment were used to classify these lines as adult plant resistant and if these data were used as a basis for broad deployment of the lines, then severe losses from stem rust would likely result, because race TKTTF is dominant in Ethiopia. The genetic mechanisms for the observed race specificity of adult plant resistance in KBG-01 and CIMMYT 18 warrant additional study. Because KBG-01 possessed the adult plant resistance gene Sr2, the observed race specificity is unexpected and may involve the presence of genes in addition to Sr2 that are regarded as non-race-specific (Singh et al. 2011). CIMMYT 18 was seedling susceptible to all races tested except JRCQC and lacked both Sr2 and Sr57/Lr34, indicating the presence of at least one unknown adult plant resistance gene.

Evaluation of field response to P. graminis f. sp. tritici races has been assessed with one race or race group only (Njau et al. 2010; Newcomb et al. 2013) or by assessing multiple races. However, previous research has generally been confounded by races being evaluated in different environments or where isolates were mixed in a bulk inoculum (Kolmer et al. 2011; Olivera et al. 2012; Rouse et al. 2011). A wheat association mapping study reported that markers significantly associated with field stem rust resistance were specific to environments where different races were assessed (Chao et al. 2017). Studies assessing the field response to multiple races in separate single-race nurseries are rare but accurately allow for the comparison of resistance responses across races, independent of the effects of different environments (Edae et al. 2018; Rouse et al. 2014b). This study was the first to evaluate the field response of emerging and virulent P. graminis f. sp. tritici races, including TTKSK and TKTTF, in separate single-race nurseries.

The single-race nursery platform in Kulumsa was successful in identifying lines with resistance effective to P. graminis f. sp. tritici races present in Ethiopia. As new virulent races emerge in the country, additional single-race nurseries can be added, or the races inoculated could be replaced. Our study demonstrated that testing of advanced breeding lines with the P. graminis f. sp. tritici races present in the country is possible and should be used to inform cultivar release and deployment.
